# DNA damage and repair in the hematopoietic system

**DOI:** 10.3724/abbs.2022053

**Published:** 2022-05-19

**Authors:** Niu Li, Hongzhu Chen, Jian Wang

**Affiliations:** 1 Department of Medical Genetics and Molecular Diagnostic Laboratory Shanghai Children’s Medical Center Shanghai Jiao Tong University School of Medicine Shanghai 200127 China; 2 Shanghai Key Laboratory of Clinical Molecular Diagnostics for Pediatrics Shanghai 200127 China; 3 Shanghai Clinical Research Center for Rare Pediatric Diseases Shanghai 200127 China

**Keywords:** c-NHEJ, DNA inter-strand crosslink, FA/BRCA pathway, hematopoietic stem cell, oxidative damage, replication stress

## Abstract

Although hematopoietic stem cells (HSCs) in the bone marrow are in a state of quiescence, they harbor the self-renewal capacity and the pluripotency to differentiate into mature blood cells when needed, which is key to maintain hematopoietic homeostasis. Importantly, HSCs are characterized by their long lifespan ( *e*. *g*., up to 60 months for mice), display characteristics of aging, and are vulnerable to various endogenous and exogenous genotoxic stresses. Generally, DNA damage in HSCs is endogenous, which is typically induced by reactive oxygen species (ROS), aldehydes, and replication stress. Mammalian cells have evolved a complex and efficient DNA repair system to cope with various DNA lesions to maintain genomic stability. The repair machinery for DNA damage in HSCs has its own characteristics. For instance, the Fanconi anemia (FA)/BRCA pathway is particularly important for the hematopoietic system, as it can limit the damage caused by DNA inter-strand crosslinks, oxidative stress, and replication stress to HSCs to prevent FA occurrence. In addition, HSCs prefer to utilize the classical non-homologous end-joining pathway, which is essential for the V(D)J rearrangement in developing lymphocytes and is involved in double-strand break repair to maintain genomic stability in the long-term quiescent state. In contrast, the base excision repair pathway is less involved in the hematopoietic system. In this review, we summarize the impact of various types of DNA damage on HSC function and review our knowledge of the corresponding repair mechanisms and related human genetic diseases.

## Introduction

Hematopoiesis refers to the process that generates blood cells of all lineages, where the cellular constituents of blood are continually replenished. Hematopoietic stem cells (HSCs) play a central role in hematopoiesis. The long-term HSCs (LT-HSCs) have self-renewal capacity and can differentiate into short-term HSCs (ST-HSCs), which are also known as multipotent progenitors (MPPs). MPPs lose the ability of self-renewal and further give rise to two types of downstream progenitors, the common myeloid progenitor (CMP) and common lymphoid progenitor (CLP). These progenitor cells then proliferate and differentiate into all types of mature blood cells [ 1, 2] . Hematopoietic homeostasis depends largely on the balance between self-renewal and differentiation of HSCs in the bone marrow (BM). Another distinguishing feature of HSCs is that they can maintain a long-term quiescent state and thus have a long lifespan (ranging from 10 to 60 months in mice) [3], where the cell cycle remains in the G0-phase without dividing [4]. Consequently, HSCs are more likely to accumulate DNA damage during the aging process. Therefore, the DNA repair machinery is important for maintaining the stability of the HSC genome, which is required for the maintenance of HSC function [ 5, 6] .

Hematopoietic cells are mainly threatened by endogenous DNA damage induced by metabolic intermediates, such as reactive oxygen species (ROS) produced by mitochondrial metabolism and aldehydes produced during lipid peroxidation. These metabolites may affect the self-renewal and differentiation of HSCs. Furthermore, they can directly induce multiple DNA lesions, including oxidative damage, DNA inter-strand crosslinks (ICLs), single-strand breaks (SSBs), and double-strand breaks (DSBs), and induce replication stress disrupting the stability of the HSCs genome [ 7– 9] . Previous studies have shown that the expression levels of most DNA damage repair genes in quiescent HSCs are downregulated compared with the downstream progenitors. Once driven into the cell cycle, the expressions of DNA damage repair genes in HSCs are increased significantly, allowing DNA breaks to be repaired stably [10]. Therefore, DNA repair defects can compromise hematopoiesis and cause bone marrow failure (BMF) or hematological malignancies [ 11, 12] .

This review comprehensively summarizes the characteristics of DNA damage and related repair mechanisms in the hematopoietic system and attempts to introduce how they affect the development and function of HSCs.

## DNA Damage and the Inducers in Hematopoietic System

Under physiological conditions, DNA damage in HSCs is mainly endogenously generated, typically induced by ROS, aldehydes, and replication stress. Here, we will review the generation mechanism, the main clearance manners, and the primary impacts on HSC development of these endogenous damage inducers.

### ROS

ROS mainly refers to hydrogen peroxide (H _2_O _2_) and superoxide anion radicals (O _2_^·−^) which are primarily produced by intracellular mitochondrial metabolism, and are also generated when cells are exposed to ionizing radiation (IR) or exogenous environmental and pharmaceutical carcinogens. ROS can be eliminated by various antioxidant defense enzymes in mammalian cells, where excessive ROS causes various DNA lesions, including abasic sites, base deamination, base oxidations, 8-oxoguanine lesions, and DNA strand breaks that disrupt the functions of DNA, RNA, and cellular proteins. One of the most studied intracellular oxidative DNA lesions is 8-oxo-7,8-dihydro-2′-deoxyguanosine (8-oxoG) which has been considered as a marker of oxidative stress [ 13, 14] .

Unlike the somatic cells that have high metabolic activity and continuously produce ROS, stem cells generally have a low metabolic rate with less production of ROS [15]. A hypoxic microenvironment and proper level of ROS in BM are essential for the self-renewal and differentiation of HSCs. Compared to the LT-HSCs from mice, ROS is increased in proliferating downstream progenitor populations, which is also true in HSCs when exiting quiescence [ 16, 17] . These results suggest that HSCs in the active cell cycle face higher levels of oxidative stress, which is consistent with the idea that HSC quiescence facilitates genomic stability [18]. Elevated levels of ROS have been considered as a characteristic of aging HSCs, and multiple evidence has shown that increased ROS impairs various functions of HSCs, including lifespan, self-renewal, and differentiation [ 8, 19, 20] .

### Aldehydes

Aldehydes, such as acetaldehyde, formaldehyde, 4-hydroxynonenal, malondialdehyde, and acrolein, represent a group of reactive metabolites which are usually resulted from the metabolism of amino acids, carbohydrates, lipids, biogenic amines, vitamins and steroids. For instance, the oxidation of endogenous methanol which is derived from the process of one-carbon metabolism in proteins, will lead to the formation of formaldehyde [21]. Moreover, alcohol drinking is also an important source of aldehydes [9]. At least 19 distinct aldehyde dehydrogenases (ALDHs) have been reported to function as detoxifying enzymes thus far [22]. The most studied members are ADH5 and ALDH2, where the metabolism of formaldehyde requires for both enzymes, while ALDH2 is more important for the metabolizing ethanol-derived acetaldehyde. Excessive aldehydes can induce various DNA lesions, including oxidative stress, DNA-ICLs, and DNA-protein crosslinks (DPCs) [ 23, 24] . As one of the most thoroughly studied types of DNA lesions, DNA-ICLs are formed by the presence of covalent adducts between two strands of DNA, which impede DNA unwinding and block DNA replication and transcription, resulting in cytotoxicity [25].

Clearance of endogenous aldehydes is critical for HSC development. Two research groups recently revealed that simultaneous loss of function of *Adh5* and *Aldh2* in mice, rather than single gene inactivation, results in elevated blood formaldehyde, and causes increased DNA damage and depletion of LT-HSCs and CLPs accompanied by growth restriction and reduced lifespan [ 26, 27] . Consistently, patients harboring bi-allelic variants in *ADH5* in combination with the rs671 defective allele of *ALDH2* were identified with BMF, myelodysplasia, and several other congenital abnormalities [ 26– 28] . The results from the patient-based induced pluripotent stem cell (iPSC) model suggest that ADH5 plays a dominant role in formaldehyde metabolism, while ALDH2 plays a secondary role [28].

### DNA replication stress

DNA replication stress is defined as the slowing or stalling of replication fork progression, which can be induced by various endogenous and exogenous sources, such as endogenous oxidative DNA lesions and DNA-ICLs, secondary DNA structures and fragile sites, R-loops, and oncogene over-expression. Replication stress is not a DNA lesion with specific structural characteristics, but rather reflects a state when the replication fork is threatened during the DNA replication process, which is more likely to cause genomic instability. In general, replicative stress produces ssDNA that binds to and is protected by replication protein A (RPA), which can recruit multiple downstream components (ATRIP, Rad17, Rad9-Hus1-Rad1 complex, and TOPBP1) to trigger the activation of the ATR/CHK1 pathway to maintain fork stability. In addition, continuous replication stress can cause replication fork stalling and collapse, and eventually leads to DSBs formation [ 29, 30] .

Endogenous DNA replication stress has been suggested as a potent driver of functional decline in aging HSCs, which has been observed from the accumulation of γH2AX foci (a widely used molecular marker for DNA damage) in HSCs from old mice; it was not caused by DNA damage, but was strongly associated with stalled and/or collapsed replication forks. In mice, old HSCs display greater levels of DNA damage associated with intracellular replication stress compared to young HSCs, accompanied by a largely decreased expression of MCM helicase components (Mcm2–7), which are pivotal for DNA replication, leading to both a delay in entering phase S and an extended S phase [18]. Consistent with the results of this study [18], the hypomorphic mice model harboring a synonymous A/G transition in exon 9 of *Atr* showed increased embryonic replication stress, accompanied by the characteristics of Seckel syndrome including accelerated aging, dwarfism, and pancytopenia [31], which have also been described in humans [32] ( Table 1).

**Table:** **Table 1** Summary of the human genetic disorders associated with DNA repair defects in the hematopoietic system

Gene	Function	Hematological phenotype in mice model	Disorders (hematologicalphenotype) in human
Key kinases
ATM	Plays a central role in DDR	BMF with HSCs depletion	Ataxia-telangiectasia (defective B cell differentiation, decreased circulating T cells)
ATR	Plays a central role in DDR	Accelerated aging, dwarfism, pancytopenia accumulation of fat in the BM	Seckel syndrome (pancytopenia)
Genes in the FA/BRCA pathway
FANCA, FANCB, FANCC, FANCE, FANCF, FANCG/XRCC9, FANCL, FANCT/UBE2T	Constituting FA core complex to promote FANCD2/I ubiquitination	Do not spontaneously develop a BMF phenotype, unless there is additional exogenous stress	FA (BMF)
FANCD2, FANCI	Recruitment of downstream nucleases to excise DNA-ICLs
FANCP/SLX4, FANCQ/ERCC4/XPF	Structure-specific exonucleases
FANCD1/BRCA2, FANCJ/BRIP1, FANCN/PALB2, FANCU/XRCC2, FANCW/RFWD3	Repair of DSB by promoting HR
FANCV/REV7/MAD2L2	Bypasss the crosslink remnants; and repair of DSB by promoting NHEJ
Genes in DSB repair machinery
DNA-PKcs/PRKDC	Serine/threonine-protein kinase that recruit interacts with LIG4-XRCC4 complex to promote DSB end ligation, and essential for V(D)J recombination	BMF with HSCs depletion	Severe combined immunodeficiency (decreased circulating T and B cells)
LIG4	ATP-dependent DNA ligase that interacts with XRCC4 to promote DSB end ligation, and essential for V(D)J recombination	Immunodeficiency, BMF with HSCs depletion	LIG4 syndrome (pancytopenia)
NBS1	A member of MRN complex, promotes DSB end resection	BMF with HSCs depletion	Nijmegen breakage syndrome (autoimmune hemolytic anemia,thrombocytopenia post hemolytic anemia, decreased circulating T and B cells)
Genes in MMR pathway
MLH1, MSH2, MSH6, PMS2	Repair of mismatches	Hematopoietic malignancies (leukemia and lymphoma	Mismatch repair cancer syndrome (leukemia)
Others
ERCC6L2	May play a role in the NER pathway	None	BMF syndrome
ADH5 and ALDH2 (Digenic)	Detoxifying enzymes that metabolize formaldehyde and acetaldehyde, respectively	Depletion of LT-HSCs and CLPs	AMED syndrome (BMF)

BM, bone marrow; BMF, bone marrow failure; CLPs, lymphoid progenitor; DDR, DNA damage response; DNA-ICLs, DNA inter-strand crosslinks; DSB, double-strand break; FA, Fanconi anemia; LT-HSCs, long-term hematopoietic stem cells; MMR, mismatch repair; MRN, MRE11-RAD50-NBS1; NER, nucleotide excision repair; NHEJ, non-homologous end joining; HR, homologous recombination; REF, reference

## DNA Repair Mechanisms in the Hematopoietic System

HSCs exhibit a certain ability to utilize the intracellular enzyme metabolism system ( *i*. *e*., ALDHs for aldehydes) to eliminate these main endogenous DNA damage inducers. However, cells still need to develop corresponding DNA damage repair mechanisms to cope with the specific damage they induce, especially when the metabolic system is insufficient to eliminate these DNA damage inducers. In the following discussions, we focus on repair mechanisms of the oxidative damage, DNA-ICL, and DSB.

### ATM-mediated oxidative damage repair

It is known that the ATM plays a critical role in responding to ROS-induced oxidative damage in HSCs. *Atm* ^–/–^ mice older than 24 months uniformly present with BMF due to defective maintenance of the HSC pool associated with increased ROS [33]. Mechanistically, the increased ROS, which might be resulted from p16 ^INK4a^-Rb pathway activation [33], can limit the lifespan of HSCs in *Atm* ^–/–^ mice through activation of p38-MAPK kinase [34]. In vitro treatment of HSCs from *Atm* ^–/–^ mice with the anti-oxidative agent N-acetyl-L-cysteine (NAC) can significantly decrease intracellular ROS levels and rescue the defective repopulating capacity [33]. Similar results were also obtained after inactivation of the p38-MAPK kinase [34].

In addition, ATM contributes to the fork head box O (FOXO) family of transcription factors-mediated ROS response. The FOXO family comprises four members (FOXO1, FOXO3, FOXO4, and FOXO6), and is another key factor in response of HSCs to oxidative DNA damage. The combined deletion of *Foxo1*, *Foxo3*, and *Foxo4* results in HSC dysfunction accompanied by increased ROS levels [35]. Specifically, FOXO3 can repress ROS by regulating the expression of ATM. *Foxo3* ^–/–^ mice display elevated ROS-related oxidative damage and defective cell cycle in HSCs, which can be restored by NAC treatment [ 36, 37] .

Failure to eliminate ROS will inevitably result in the generation of DSBs. It is worth noting that ATM also plays an important role in the repair of ROS-induced endogenous DSBs [38], which may contribute to the maintenance of HSCs. Details will be discussed in the “HR and c-NHEJ to repair DSB” section.

### Fanconi anemia (FA) pathway to repair DNA-ICLs

In addition to the endogenous aldehydes, DNA-ICLs are also commonly formed upon the application of exogenous chemotherapy drugs, such as chlorambucil, mitomycin C (MMC), and platinum compounds [39]. The repair of DNA-ICL has a special clinical significance due to its association with the occurrence of FA. FA is a rare genetic disease with an estimated incidence of 1–5/1,000,000 live births and is characterized by progressive BMF, congenital abnormalities, and an increased incidence of cancers. Consequently, cells derived from FA patients show hypersensitivity and increased chromosome abnormalities which are typically characterized by radial aggregation of chromosomes and chromosome breakage, upon treatment with DNA-ICL agents ( *e*. *g*., MMC) [ 40, 41] .

**Figure 1:**
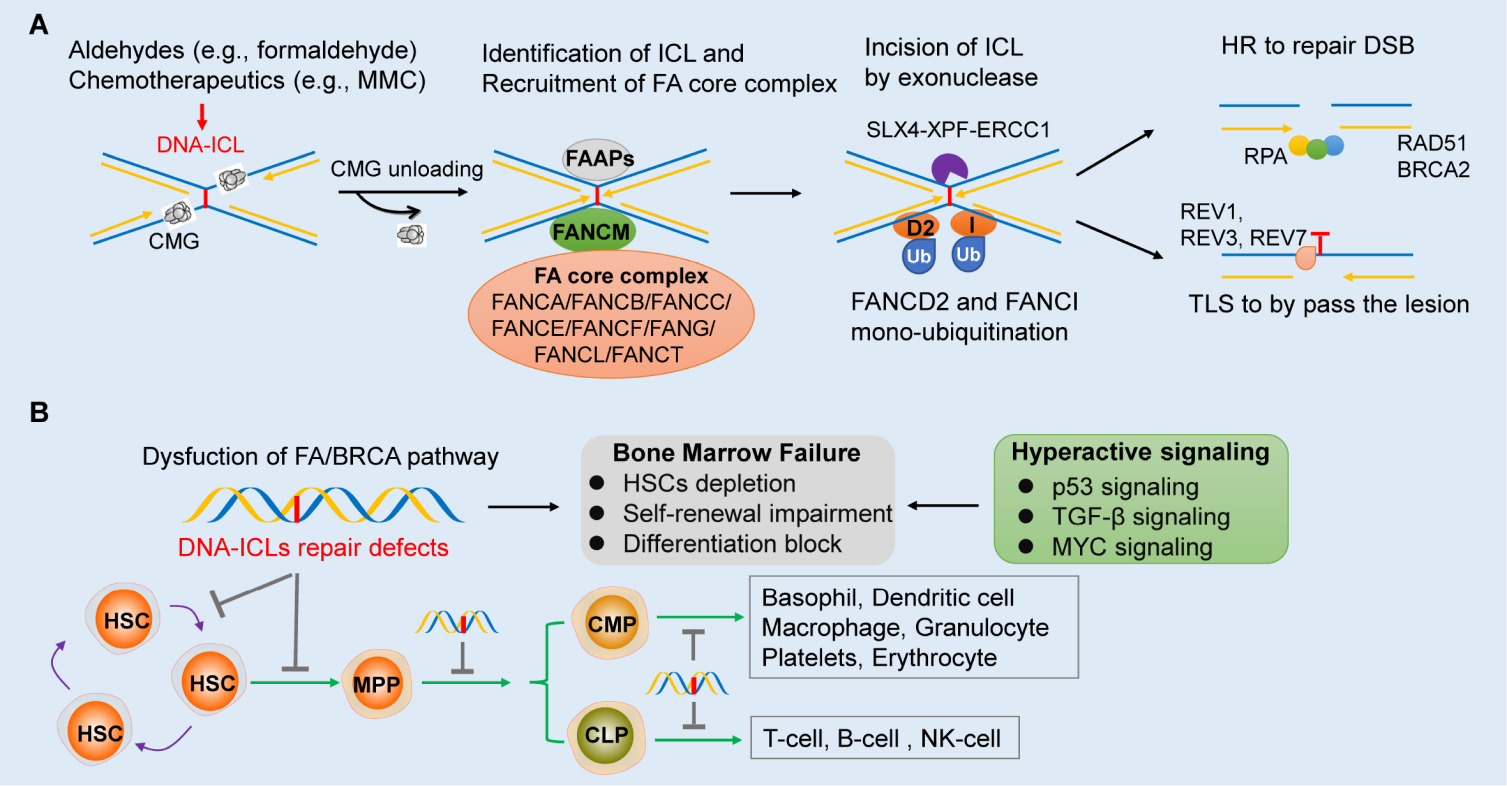
Figure 1 The FA/BRCA pathway and HSC development (A) Brief description of the DNA inter-strand crosslink (ICL) repair mechanism by the FA/BRCA pathway. DNA-ICLs induced by various exogenouschemotherapeutics ( e. g., MMC) or endogenous aldehydes ( e. g., formaldehyde) are first recognized by FANCM after the CMG (CDC45/MCM2-7/GINS) complex dissociation from the replication fork. FANCM then acts as a bridge to recruit other core complex members to ICLs to catalyze the mono-ubiquitination of FANCD2 and FANCI. Ubiquitinated FANCD2/FANCI can recruit the SLX4-XPF-ERCC1 exonuclease complex to unhook DNA-ICLs with the generation of DSBs. Lastly, DSBs are further repaired by HR, and the crosslink remnants are bypassed by TLS. (B) In the hematopoietic process, the HSCs must maintain self-renewal to prevent depletion of the stem cell pool in the BM. In addition, the HSCs have the pluripotency to differentiate into MPPs that can further differentiate into CMPs and CLPs and the downstream mature blood cells when needed. Defects in the FA/BRCA pathway will result in DNA-ICLs repair defects and genomic instability of HSCs, leading to bone marrow failure with HSCs depletion, self-renewal impairment, and differentiation block. FA, Fanconi anemia; FAAPs, FA-associated proteins; MMC, mitomycin C.

The FA pathway, also known as FA/BRCA pathway, plays a dominant role in the DNA-ICL repair, consisting of 22 key FANC proteins (FANCA-W) and many associated proteins ( *e*. *g*., FAAP10, FAAP16, FAAP20, FAAP24, FAAP100, and FAN1). Of the FANC genes, mutations in 18 *bona fide* genes lead to the occurrence of FA ( Table 1), while the other 4 FANC genes ( *FANCM*, *FANCO*/ *RAD51C*, *FANCR*/ *RAD51*, and *FANCS*/ *BRCA1*) are associated with FA-like syndrome [41]. The FA/BRCA pathway for DNA-ICL repair is extremely complex, and can be briefly summarized with the following 5 steps ( Figure 1A): (1) The CMG helicase complex (Cdc45/MCM2-7/GINS) is unloaded from the stalled replication fork, allowing the leading strand to approach the ICL; (2) FANCM, a member of the FA core complex (FANCA/FANCB/FANCC/FANCE/FANCF/FANG/FANCL/FANCT), recognizes the ICL and acts as a “bridge” to recruit other members of the core complex to catalyze the mono-ubiquitination of FANCD2 and FANCI; (3) Ubiquitinated FANCD2-I recruits the structure-specific exonuclease (SLX4-XPF-ERCC1) to unhook the ICL with the generation of a DSB; (4) RAD51-dependent HR to resolve the DSB (details can be seen in the next section “HR and c-NHEJ to repair DSB”); and (5) REV1-REV3-REV7 (Polζ)-dependent translesion synthesis (TLS) to bypass the crosslink remnants [ 40, 42, 43] . This process requires the E3 ubiquitin ligase FANCW (also known as RFWD3) to mediate PCNA ubiquitylation, which is an essential step for TLS polymerase recruitment [44].

Dysfunction of the FA/BRCA pathway results in genome instability and causes HSCs developmental defects ( Figure 1B). Reduction in the number, blocked differentiation, and compromised self-renewal of HSCs have been widely observed in the FA-deficient HSCs [ 45– 47] . Recently, Shen et al. [48] uncovered that elevated nuclear formaldehyde generated during hematopoietic differentiation is the endogenous genotoxin causing blood attrition in FA patients. Cellular formaldehyde appears to be necessary for blood progenitor/stem cell differentiation, since the formaldehyde levels of *FANCL* ^–/–^ cells are comparable to those of wild-type cells under V-D3 induction. However, differentiating cells display higher levels of formaldehyde-inflicted DNA-ICLs in the absence of *FANCL*, and thus encounter a significantly elevated level of genotoxic stress, resulting in defects in cell differentiation *in vitro*. In addition, systematic studies of human FA cell lines and BM samples, including genome-wide shRNA screening and single-cell RNA sequencing (scRNA-seq) strategies, reported that several signaling pathways are closely related to the occurrence and development of FA. These include the p53, transforming growth factor-β (TGF-β), and MYC signaling pathways ( Figure 1B). In BM and cell samples from FA patients, unrepaired DNA damage induces continuous hyper-activation of p53/p21 *^CDKN1A^*, resulting in G0/G1 cell-cycle arrest. Depletion of p53 can rescue hematopoietic stem/progenitor cells (HSPCs) defects in *Fancg* ^–/–^ mice and improve the clonogenicity of CD34 ^+^ cells [49]. Zhang *et al*. [50] reported that the TGF-β pathway is significantly upregulated in FA/BRCA pathway-deficient cells, which has also been observed in myelodysplastic syndrome [51], myelofibrosis [52], and Shwachman-Diamond syndrome, another inherited BMF syndrome (IBMFS) [53]. Hyperactive TGF-β signaling impairs FA cell survival following MMC treatment. Inhibition of TGF-β signaling can rescue the dysfunction of HSCs derived from humans and mice [50]. Rodríguez *et al*. [54] revealed that MYC is overexpressed in FA samples, which can promote the proliferation of FA HSCs, but with a much higher level of DNA damage, and cause subsequent exhaustion of HSCs in FA BM. Thus, MYC inhibition decreases HSC proliferation; however, it can reduce physiological and genotoxic stress in HSCs from *Fancd2* ^–/–^ mice.

Although the importance of the FA/BRCA pathway in maintaining the genome stability in human HSPCs has been well established, it has encountered great challenges when using murine models to further explore the molecular mechanisms, mainly because mice with FA/BRCA pathway defects do not naturally develop BMF. One possible explanation is that mice are not under constant physiological stress as human HSCs are [17]. Indeed, treatment of *Fanca* ^–/–^ mice with polyinosinic:polycytidylic acid (pI:pC) impairs the capacity to re-enter the long-term quiescent state and repopulation activity of the HSCs, and eventually causes BMF [17]. Consistent with this idea, combined deletion of the FA gene with *Adh5* or *Aldh2* in mice can induce DNA damage accumulation in BM cells and cause BMF [ 55, 56] . Therefore, the induction of BMF in FA gene-deficient mice through appropriate exogenous stress stimulation has been widely used to study the pathogenesis of FA.

It should be noted that how the defects in the molecular mechanism of FA/BRCA pathway leads to the occurrence of FA or other types of IBMFSs has not been fully explored. For example, Hodskinson *et al*. [57] reported a novel acetaldehyde-induced DNA-ICL repair pathway mediated by REV1, which requires fork convergence, but not CMG unloading. Unlike the FA/BRCA pathway, this incision-free pathway does not produce DNA strand breaks or abasic sites, thus avoiding large-scale genome instability. Notably, depletion of *REV1* sensitizes cells to MMC and cisplatin, resulting in increased chromosome breakage and radial formation [58]. However, no *REV1* variants have been reported in patients with FA or other hematopoietic abnormalities. It will be interesting to investigate whether digenic variants in *REV1* and *ALDHs* contribute to the BMF phenotype in the future. In addition, novel FA/BRCA pathway regulators that function in DNA-ICL repair have been identified. Although the loss of function of some genes, such as *NIPA* [59] and *NLRP12* [60], has been linked to BMF in mice, their molecular mechanisms for repairing DNA-ICLs or their impacts on HSC function and phenotype in humans have not been fully elucidated. Conversely, some genes have shown definitive functions in DNA-ICL repair; however, their roles in hematopoiesis have not been explored, such as *SAN1* [61] and *NEIL3* [ 62, 63] .

### FA/BRCA pathway to repair oxidative damage

The FA/BRCA pathway plays a critical role in the repair of oxidative damage in the hematopoietic system. It has been suggested that the occurrence of FA is not only caused by DNA-ICL repair defects, and oxidative stress is also involved in the pathogenesis of FA. Early evidence is arised from the following: (1) FANCC interacts with NADPH cytochrome-P450 reductase (RED) [64], and (2) FANCG interacts with and suppresses the activity of cytochrome P450 2E1 (CYP2E1) in DNA oxidation [65]. This idea is corroborated by the increasing evidence from both human and mouse FA models. For example, *in vitro* treatment of FA patients’ cells with H _2_O _2_ resulted in similar effects to ICL inducers, including decreased colony formation of the BM progenitor cells and G2/M arrest of the lymphoblasts. Mechanistically, FA can prevent the promoters of antioxidant defense genes from being damaged by oxidative stress, thereby maintaining their expression levels [66]. In mice, double knockouts of *Fancc* and cytosolic Cu/Zn superoxide dismutase ( *Sod1*) cause a series of blood cell abnormalities, including bone marrow hypocellularity, erythrocytopenia, and leucopenia [67]. Consistently, *Fancc* ^–/–^ hematopoietic progenitors are hypersensitive to H _2_O _2_, and the anti-oxidative agent NAC can improve the survival of *Fancc* ^–/–^ murine embryo fibroblasts [68].

### FA/BRCA pathway to protect replication forks

Increasing evidence shows that the FA/BRCA pathway is involved in replication fork protection and the restart of stalled replication forks, suggesting that replication stress may contribute to the occurrence of FA. BRCA1, BRCA2, and RAD51 have shown a strong ability to protect stalled replication forks from the nucleolytic degradation independent of DSB repair. Additionally, FANCA, FANCD2, and FANCM exhibit the ability to protect the replication forks [ 69– 72] . In response to replication fork stalling, RPA is polyubiquitinated by FANCW and facilitates the restart of stalled replication forks [73]. In addition, the FA/BRCA pathway directs break-induced replication (BIR) machinery to promote stalled replication forks involved in fork cleavage by SLX4 and FAN1 endonucleases [ 74, 75] . Interestingly, although extensive evidence confirms that the FA/BRCA pathway is directly involved in the protection of replication forks under replication stress, most FA-deficient cells seem to be resistant to replication stress agents, such as hydroxyurea (HU) [76]. Xu *et al*. [75] demonstrated that the FA-deficient cells are hypersensitive to replication stress only when persistently treated with low doses of HU or aphidicolin for two to three weeks. Consistently, daily injection of HU into *Fancl*-deficient adult mice for six weeks resulted in BMF. Several negative regulators of replication fork protection, including the DNA/RNA helicase SLFN11 [77] and LNK/SH2B3 [78], have recently been identified that may contribute to the attrition of HSCs in FA. The high expression of SLFN11 in HSCs promotes fork degradation in PD20 (FANCD2 ^–/–^) cells. Depletion of SLFN11 in PD20 cells can prevent replication fork degradation and rescue the FA phenotype of cells [77]. Similarly, depletion of *Lnk* restores HSC function in *Fancd2* ^−/−^ mice [78]. These new findings once again indicate that replication stress is an important cause of HSCs dysfunction in FA patients and has strong therapeutic significance.

### HR and c-NHEJ to repair DSB

**Figure 2:**
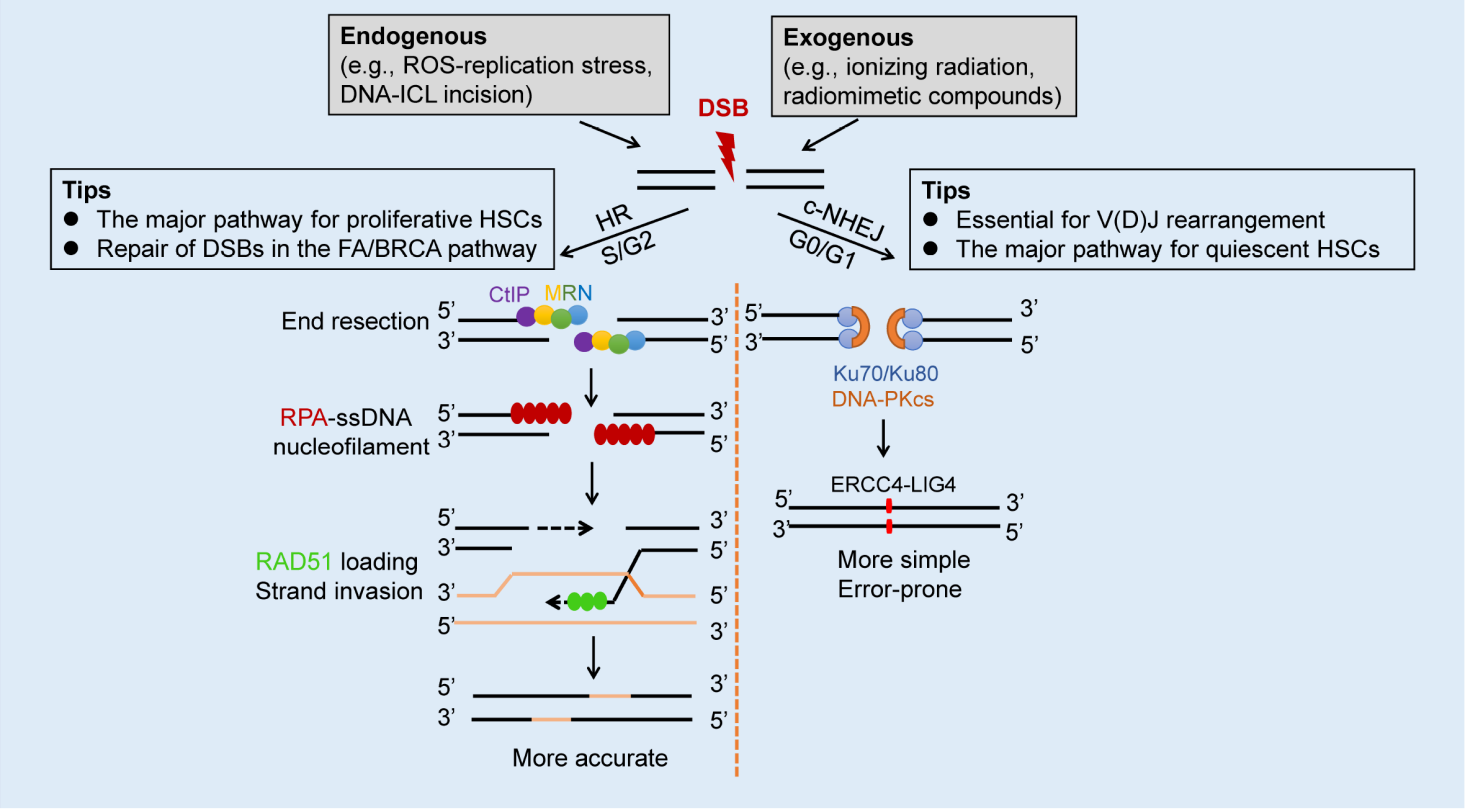
Figure 2 Brief description of the DSB repair mechanism in hematopoietic system In HSCs, DSBs are mainly caused by excessive endogenous replication stress ( e. g., ROS-induced) and DNA-ICL incision or application of exogenous chemotherapeutics ( e. g., ionizing radiation and radiomimetic, compounds). Two major pathways, termed HR and c-NHEJ facilitate DSB repair. HR mainly occurs in the S/G2-phase, where the DSB ends first undergo nucleolytic resection performed by the CtIP-MRN (MRE11-RAD50-NBS1) complex to generate single-strand DNA (ssDNA). The subsequent steps include replication protein A (RPA)-ssDNA nucleofilament formation, RAD51 loading to replace RPA, and strand invasion. The proliferative HSCs mainly use HR to complete DSB repair, which is also true for the repair of the DSBs generated in the FA/BRCA pathway. c-NHEJ repairs DSBs in the G0/G1-phase, where the DNA ends are blocked by Ku70/Ku80/DNA-PKcs and then undergo blunt end ligation by XRCC4-LIG4. It is well known that c-NHEJ is essential for V(D)J rearrangement during lymphocyte development. In addition, c-NHEJ plays the dominant role in DSBs repair for quiescent HSCs, which are in the G0-phase of the cell cycle. HSC, hematopoietic stem cell; ssDNA, single-strand DNA.

Endogenous DSBs in the hematopoietic system mainly arise from the collapsed replication forks and the incision of the aldehyde-induced DNA-ICLs by the FA/BRCA pathway. In addition, HSCs are under stress from exogenous DSBs, especially when patients with malignant tumors are treated with IR, radiomimetic compounds ( *i*. *e*., bleomycin), topoisomerase inhibitors ( *i*. *e*., camptothecin and etoposide), and ribonucleotide reductase inhibitors ( *i*. *e*., HU) [ 7, 79] . As the most cytotoxic type of DNA lesion, the repair of DSBs in vertebrate cells is mainly accomplished by the HR and classical non-homologous end-joining (c-NHEJ) pathways, and there are stricter cell cycle restrictions: c-NHEJ mainly occurs in the G0/G1 phase, while HR mainly functions in the S/G2- phase [80]. c-NHEJ is error-prone because the DSB end is blocked by Ku70/80 and DNA-PKcs, and can only be repaired by blunt end ligation by LIG4, independent of sequence homology. Ligation is catalyzed by LIG4, which is recruited to the DNA ends by XRCC4 ( Figure 2). In contrast, HR is usually error-free with the use of a sister or homologous chromatid as a template. DSBs generated from DNA-ICLs incision by the FA/BRCA pathway are mainly repaired by HR; therefore, cells from FA patients exhibit high c-NHEJ activity, leading to chromosomal abnormalities.

Mounting evidence has shown that the c-NHEJ pathway is essential for hematopoiesis, especially for V(D)J rearrangement in developing lymphocytes [81]. Accordingly, HSCs from *DNA*- *PKcs* ^–/–^ and *Ku80* ^–/–^ mice did not develop into mature B and T cells [ 82, 83] . In addition, the mice with mutations at the phosphorylation sites of *DNA*- *PKcs* (DNA-PKcs ^3A/3A^) died early due to congenital BMF [84]. *Ku80* ^–/–^ and the hypomorphic Lig4 ^Y288C^ mutant mice display HSC defects under stress conditions, which are particularly evident in aging HSCs [ 85, 86] . Consistently, bi-allelic germline variants of *DNA-PKcs* and *LIG4* in humans have been reported to cause severe combined immunodeficiency (decreased circulating T and B cells) [87] and LIG4 syndrome (pancytopenia) [88], respectively ( Table 1). These results are consistent with the fact that HSCs tend to utilize c-NHEJ rather than HR to maintain genomic stability. HSPCs from mice have a higher baseline level of NHEJ and show resistance to low doses of IR compared to CMPs. Irradiated HSPCs showed cell cycle arrest, whereas CMPs showed cell death. Further studies suggested that quiescent HSPCs use error-prone NHEJ to complete IR-induced DSBs for survival, and the remaining surviving CMPs use HR to repair DSBs. Interestingly, HR activity was significantly increased after IR treatment in proliferative HSPCs, indicating that the quiescent state of HSPCs greatly limits their ability to use high-fidelity HR [89].

The key event that determines the path of DSB repair is the end resection of DNA. DSBs without end resection can only be repaired by c-NHEJ, and once the DSB undergoes end resection, it is mainly repaired by HR [ 80, 90] . The initial stage of DSB end resection is mediated by the nuclease CtIP and MRN complex (MRE11-RAD50-NBS1). In the second stage of extensive resection, more helicases and exonucleases, including DNA2, BLM, WRN, and EXO1, are required to cut the DSB into single-stranded DNA (ssDNA) [ 91, 92] . RPA is immediately recruited to ssDNA and subsequently replaced by RAD51 ( Figure 2). The formation of ssDNA-RAD51 nucleofilaments is essential for mediating the formation of the D-loop of DNA strands [93]. ssDNA-RPA not only protects the DNA ends from being degraded after resection, but also recruits ATR and triggers the ATR signaling pathway (CHK1 phosphorylation) to ensure that HR repair proceeds smoothly in the S/G2 phase [ 80, 94] .

The MRN complex recruits and activates ATM, whose activation plays a central role in DSB repair signaling by phosphorylatingseveral downstream effectors, such as BRCA1 and ATR. In addition to the crucial role in DSB repair, MRN complex may also help HSCs face replication stress by directly promoting the restart of stalled replication forks or by suppressing R-loop formations at transcription-replication conflicts [ 95– 97] . In line with this hypothesis, hematopoietic abnormalities have been observed in mice models deficient of *Atm* [ 33, 34] , *Mre11* [98], *Rad50* [99], and *Nbs1* [ 100, 101] , respectively. In addition, *ATM* and *NBS1* have been associated with hematopoietic phenotypes in humans, and bi-allelic variants of each gene can lead to defects in T and B cells [ 102, 103] ( Table 1).

Recently, Shao *et al*. [104] revealed that DNA-PKcs plays an rRNA-dependent role in hematopoiesis, which is independent of its classical function in c-NHEJ. Defects of phosphorylation at the T2609 cluster of DNA-PKcs impair 18S rRNA processing, cause global translation defects in hematopoietic cells, and ultimately lead to BMF in mice. Similarly, γH2AX, a sensitive molecular marker of DSBs, which is phosphorylated by ATM, ATR, and DNA-PKcs kinases on serine 139, was also found to have functions beyond the DNA damage response [105]. γH2AX can transcriptionally inhibit rDNA genes, leading to impairment of ribosome biogenesis in quiescent old HSCs [18]. These indicate that the regulation of ribosome biogenesis by classical DNA damage/DNA repair genes may present a new study topic for HSC aging and BMF.

### BER, MMR, and NER in hematopoiesis

The base excision repair (BER), DNA mismatch repair (MMR), and nucleotide excision repair (NER) pathways are the three excision repair pathways that primarily resolve single-stranded DNA damage. Little is known about BER and hematopoiesis, hence we focus on MMR and NER in this section. The MMR pathway is used for the repair of mismatches which mainly arise from replication errors when confronted with base pair anomalies, such as O ^6^-mG and 8-oxoG [106]. MMR defects are associated with microsatellite instability (MSI), a marker of genomic instability, which is frequently observed in colorectal cancer [107]. In humans, the MMR pathway consists of seven members, including MLH1, MLH3, MSH2, MSH3, MSH6, PMS1, and PMS2 [ 108, 109] . HSCs from older individuals (>45 years old) display greater methylation in the *MLH1* promoter compared to HSCs from younger individuals (<45 years old), leading to lower MLH1 expression and increased MSI [110], suggesting that MLH1-mediated MMR plays an important role in maintaining genomic instability of HSCs during aging. Defects in the MMR pathway will drive HSC malignancy and lead to hematopoietic malignancies [111], whereas BMF is rare in patients with MMR deficiency. Indeed, bi-allelic germline variants in *MLH1* have been reported in the early onset of leukemia and lymphoma [112]. Moreover, *Mlh1* ^+/-^mice show an increased incidence of lymphomagenesis upon simulated space radiation exposure [113]. Similar evidence can be observed in both patients and (or) mouse models with defects in *MSH2*, *MSH6*, and *PMS2* [ 112, 114, 115] ( Table 1).

NER is particularly important for the repair of ultraviolet light-induced lesions, such as thymine dimers and 6,4-photoproducts. Thus, NER defects are strongly associated with the risk of developing skin cancer. Germline variants of classic genes ( *e*. *g*., *ERCC2*/ *XPD*, *ERCC3*/ *XPB*, *ERCC4*/ *XPF*, *ERCC5*/ *XPG*, *ERCC6*/ *CSB*, and *ERCC8*) in the NER pathway frequently cause Xeroderma pigmentosum, trichothiodystrophy, or Cockayne syndrome [116]. Hematopoietic abnormalities are rarely associated with NER, except for *ERCC4*/ *XPF*, which has been linked to FA [117]. Nevertheless, evidence does show that NER is involved in hematopoietic processes. For instance, although the establishment, maintenance, or expansion of LT-HSCs during aging is normal, the numbers of CMPs and CLPs in *XPD*-deficient mice are significantly reduced [86]. Moreover, ERCC6L2 is reportedly associated with NER, as depletion of ERCC6L2 causes mild sensitivity to MMC and Irofulven, but not to topoisomerase inhibitors. Bi-allelic *ERCC6L2* variants have been reported in multiple BMF patients [ 118, 119] .

## Conclusions and Perspectives

This review attempts to classify the DNA damage that blood cells may encounter and specifically describe the repair mechanisms involved in each type of DNA damage. These DNA lesions, as well as the repair pathways, are not isolated from each other, which is reflected not only in the fact that a single DNA damage may require multiple repair mechanisms, but also in the fact that a single repair mechanism plays a role in multiple types of DNA damage. For example, DNA-ICL and ROS can cause replication stress, and if the replication stress cannot be resolved in time, the replication fork may collapse and generate DSBs. The classic FA/BRCA pathway requires the cooperation of HR to repair DNA-ICLs because DSBs are inevitably produced in this process.

Due to the difficulty in obtaining human HSCs, most studies have been conducted in mice. Given that FA-knockout mice do not spontaneously develop a BMF phenotype, additional exogenous stress, such as pI:pC, aldehydes, and HU treatment should be considered to test the hematopoietic phenotype when studying the pathogenesis of a novel candidate FA gene. In addition, several DNA repair genes have been shown to be important for HSC function, but only a few have been validated in humans ( Table 1). For example, no FA patients harbor germline variants in FA-associated genes, which are essential for ICL repair. Moreover, HSC dysfunction has been demonstrated in *Ku80* ^–/–^ mice [86], but related phenotypes ( *e*. *g*., BMF or blood cell reduction) have not been reported in humans. Exploring the underlying mechanisms of these phenomena is essential for understanding the pathogenesis of related diseases and may also provide opportunities for the development of potential treatments.
